# The Mediating Role of Health Consciousness in the Relation Between Emotional Intelligence and Health Behaviors

**DOI:** 10.3389/fpsyg.2018.02161

**Published:** 2018-11-08

**Authors:** Adriana Espinosa, Selma Kadić-Maglajlić

**Affiliations:** ^1^Colin Powell School for Civic and Global Leadership, Department of Psychology, The City College of New York, New York, NY, United States; ^2^School of Economics and Business, Department of Marketing, University of Sarajevo, Sarajevo, Bosnia and Herzegovina

**Keywords:** health consciousness, emotional intelligence, health behaviors, latent class analysis, mediation

## Abstract

The goals of this study were to identify groups of health-related behaviors among young adults (*N* = 314, *M*age = 21.94, *SD* = 6.53), gauge the relation between emotional intelligence and health behaviors in this population, and assess health consciousness as mediator of said relation. Latent class analysis identified two mutually exclusive health behavior groups, which according to response patterns were labeled as Healthy and Unhealthy. The Healthy group (56%) was composed of individuals who had a healthy diet (i.e., low fat and high fiber), exercised regularly, and who frequently engaged in behaviors that prevent oral and skin-related diseases. In contrast, the Unhealthy group (44%) rarely engaged in these health-promoting behaviors. Using structural equation modeling we found a negative relation between emotional intelligence and unhealthy behaviors relative to health-promoting ones. Mediation analyses indicated that the mechanism explaining said relation was through increments in health consciousness, with large standardized indirect effects ranging between -0.52 and -0.78. As health behaviors during early adulthood are salient predictors of health outcomes in old age, the results have clear implications for the inclusion of emotional intelligence training in programs seeking to raise health awareness and cultivate health promoting behaviors in young adults, in so much as to seek to reduce the risk of chronic ailments later in life.

## Introduction

Studies show that prolonged participation in poor health practices such as unhealthy eating, low physical activity, and noncompliance with disease detection and prevention guidelines are salient markers of adverse physical health conditions later in life (Rimm et al., 1995; Colditz et al., 2000; Patel et al., 2010; Chiuve et al., 2012). In particular, unhealthy eating and low physical activity promote obesity, which is a major contributor for diabetes, heart disease, and other chronic health problems in later years (Must et al., 1999; Kopelman, 2000; Bogers et al., 2007). Long-term smoking and excessive alcohol use increase the risk of developing heart and pulmonary disease as well as multiple forms of cancer (U.S. Department of Health and Human Services, 2010). Failure to participate in disease screening and prevention practices increase the risk of developing multiple illnesses and additional health complications later in life (Ajwani et al., 2003; Yach et al., 2004; Adolph et al., 2017). Therefore, research identifying the factors related to health-promoting behaviors is vital for creating interventions aimed at reducing the risk of illness, particularly among young adults whose health behaviors are susceptible to change (Frech, 2012; Johnstone and Hooper, 2016; Daw et al., 2017) and will have important health implications in late adulthood (Anderson and Horvath, 2004; National Research Council and Institute of Medicine of the National Academies, 2009; Park et al., 2014). Two psychological concepts, emotional intelligence and health consciousness, have been identified as important predictors of health behaviors.

Emotional intelligence (EI), a concept derived from principles of social intelligence (Petrides, 2011), refers to a person’s emotional competence in social interactions, particularly the perception, understanding, expression, use and regulation of feelings and emotions (Petrides and Furnham, 2001; Wong and Law, 2002; Johnson et al., 2009; Nelis et al., 2011). Substantial Meta-analytic evidence has documented EI as a positive predictor of physical and mental health (Schutte et al., 2007; Martins et al., 2010), and related studies have found EI to be correlated with health behaviors (Petrides et al., 2016). In particular, studies have shown that individuals with high EI actively participate in health-promoting behaviors, such as following a healthy diet as well as engaging in physical activity (Saklofske et al., 2007a,b; Fernández-Abascal and Martín-Díaz, 2015; Mikolajczak et al., 2015). Studies have also found that low EI is associated with the use of health-impairing substances, including alcohol, tobacco, and illicit drugs (Trinidad and Johnson, 2002; Riley and Schutte, 2003; Brackett et al., 2004; Trinidad et al., 2004). In sum, EI is a widely documented construct that relates to improved health and related behaviors.

Health consciousness corresponds to self-awareness about one’s health, and the willingness to engage in health and wellness promoting behaviors (Gould, 1988, 1990; Michaelidou and Hassan, 2008). Not surprisingly, health conscious individuals actively seek for information about how to improve their health, and adhere accordingly (Iversen and Kraft, 2006; Dutta and Feng, 2007; Basu and Dutta, 2008). Hence, individuals with high health consciousness have positive attitudes about nutrition, self-care and exercise, and accordingly have healthier lifestyles than individuals with low health consciousness (Hollis et al., 1986; Kraft and Goodell, 1993; Hoek et al., 2004; Chen, 2009). Such healthier lifestyles also include frequently visiting a primary doctor, and having a lower propensity to engage in prescription drug misuse (Mesanovic et al., 2013; Lucas et al., 2017).

Despite numerous evidence highlighting EI and health consciousness independently as important markers of behaviors that promote health and well-being, to our knowledge no research has considered the interrelation between these psychological concepts in predicting health behaviors. We posit that EI relates to health behaviors indirectly through increases in health consciousness for two primary reasons. First, high EI individuals are motivated to adjust behavior for the sake of improving their overall well-being and achieve success (Goleman, 1998). Accordingly, high-EI individuals develop high levels of self-awareness and appraisal, including awareness about behaviors that promote health (Wong and Law, 2002). Second, high EI individuals possess the capacity for emotion regulation (Dawda and Hart, 2000; Peña-Sarrionandia et al., 2015), and accordingly develop a heightened awareness of effective and healthy strategies to manage daily life stressors (Barrett et al., 2001; Peña-Sarrionandia et al., 2015). In sum, EI is a potential precursor of health consciousness, which in turn relates to actions taken to improve one’s health, thus mediating the relation between EI and health behaviors.

### Current Study

In this article, we test the mediating role of health consciousness in the EI to health behaviors relation. Specifically, we hypothesized that EI and health consciousness would negatively relate to unhealthy behaviors. Moreover, we hypothesized that the relation between EI and health behaviors would be indirect through changes in health consciousness.

## Materials and Methods

### Procedure and Participants

Data were obtained from a large and culturally diverse public university in the Northeastern United States. Participants were prescreened for English proficiency, age, and any diagnosed physical or mental illness. A total of 314 healthy adults (18 years or older) completed self-report questionnaires in a research laboratory setting. The average participant was 21.94 (*SD* = 6.53) years old. Participants were mostly female (62.4%), and racial ethnic minority (85.0%), including Black (15.3%), Hispanic (33.4%), Asian (28.3%), and multi-racial (8.0%). The majority of participants (61.8%) reported household incomes of $40,000 USD or lower, which falls below the median household income of the city (United States Census Bureau, 2016). The vast majority reported their physical health between excellent and fair at time of participation (97.5%), with only eight participants reporting poor physical health. The majority of the sample had a primary doctor they visited at least every 6 months (79.0%), as well as a dentist (69.1%). The Institutional Review Board of the university approved this study, and all participants provided written consent.

### Measures

#### Emotional Intelligence

Participants answered the Wong & Law Emotional Intelligence Scale (WLEIS; Wong and Law, 2002), which is a 16-item self-report measure of EI that is based on the four-branch ability model (Salovey and Mayer, 1990; Mayer and Salovey, 1997), and which has been identified as a theoretically supported measure of EI that correlates with personality traits (Brannick et al., 2009). Specifically, the measure gauges four dimensions of emotional intelligence including, emotional appraisal and expression of one’s emotions (SEA), emotional appraisal and recognition of other’s emotions (OEA), self-regulation of emotions (ROE) and use of emotions to enable performance (UOE). Combined, these dimensions provide a global measure of EI. Sample items include “I have a good sense of why I have certain feelings most of the time” and “I am a self-motivated person.” Items are presented using a 7-point Likert Scale (1 = Strongly Disagree … 7 = Strongly Agree). The psychometric properties of the WLEIS scale have been well-documented in multiple cultures and ethnic groups within and outside the US (Law et al., 2004; Ng et al., 2008; Li et al., 2012; Carvalho et al., 2016).

#### Health Consciousness

Respondents also answered questions from the Health Consciousness Scale (HCS; Gould, 1988), which is a 9-item self-report global measure of one’s health awareness. Sample items include “I reflect about my health a lot” and “I am alert to changes in my health.” Items are presented on a 7-point Likert scale (1 = Strongly Disagree … 7 = Strongly Agree). The scale has been validated in studies using international as well as US-based samples (Gould, 1990; Bearden et al., 2011; Mesanovic et al., 2013).

#### Health Behaviors

We assessed health behaviors via the Health Behavior Schedule II (HBS-II; Heiby et al., 2005; Frank et al., 2007), a self-report measure of acquiescence to conventional health practices that has been documented to predict compliance (Frank et al., 2007). While the original scale contains 12 items, only 9 items were used in this study, as these were most relevant for our population. Individuals were asked to state the degree to which they have succeeded (1 = Not at successful… 7 = Very successful) in eating a healthy diet, regularly exercising, flossing teeth daily, protecting skin from sun daily, refraining from smoking and drinking alcohol, taking medications as prescribed, performing a monthly breast exam, and screening for cervical/prostate cancer every 3 years. The reliability of the scale has been assessed in the literature (Frank et al., 2007).

#### Covariates

Respondents provided additional information including their age in years, sex (Male or Female), and family income (1 = $0 – $20,000 … 5 = $80,000 and above).

### Analytical Strategy

Confirmatory Factor Analysis (CFA) with maximum likelihood estimation using the sample covariance matrix as input assessed the psychometric properties of the EI and health consciousness scales. Measures of absolute and relative fit, including the χ^2^ statistic, root mean square error of approximation (RMSEA), the comparative fit index (CFI), the Tucker Lewis Index (TLI), and the standardized root mean squared residual (SRMR) determined goodness of fit. In particular, a good fitting model is determined by fit measures that adhere to the following benchmarks: *p*-value of χ^2^ statistic > 0.05, RMSEA ≤ 0.06, CFI ≥ 0.95, TLI ≥ 0.95, and SRMR ≤ 0.09 (Hu and Bentler, 1999). Convergent validity of each construct was determined by comparing each construct reliability (CR) estimate against a minimum benchmark value of 0.70 and by verifying that each average variance extracted (AVE) was greater than 0.50 (Fornell and Larcker, 1981; Bagozzi and Yi, 1988). In addition, for each construct, discriminant validity was determined by comparing the AVE to the constructs’ squared correlation (Fornell and Larcker, 1981).

According to the literature, health behaviors are more likely to appear in groups or clusters than they are to appear independently (Vickers et al., 1990; Daw et al., 2017). However, the different types of health behavior groupings characterizing our population are unknown. Therefore, we used latent class analysis (LCA) to determine the patterns of health behaviors evident in our sample. Latent class analysis is a finite mixture model that probabilistically sorts respondents into several mutually exclusive groups with similar response patterns (McCutcheon, 1987; Hagenaars and McCutcheon, 2002). Thus in this setting, LCA generated a categorical outcome variable representing different types of health behavior groups. Because each health behavior question had seven possible responses (i.e., 1 = Not at successful… 7 = Very successful), we employed polytomous LCA (Linzer and Lewis, 2011). The number of groups that define the best fitting LCA model corresponds to those that minimize the Bayesian Information Criterion (BIC; Nylund et al., 2007; Tein et al., 2013) and the Consistent Akaike Information Criterion (CAIC; Akaike, 1973; McCutcheon, 1987). In addition, a good fitting model will yield an entropy index close to 1, representing clear demarcation of the classes (Ramaswamy et al., 1993).

Mediation analyses using structural equation modeling assessed the relation between emotional intelligence and health behaviors through health consciousness. Goodness of fit measures as mentioned above for CFA were adhered to. The 95% confidence interval for the indirect effect via health consciousness was obtained using 5,000 bootstrapped replications. Mediation is confirmed if such confidence interval does not contain zero (Shrout and Bolger, 2002; Hayes, 2013). All models included age, sex, and family income as covariates. We report partially and completely standardized indirect effects as measures of effect size in mediation models (Preacher and Kelley, 2011).

Pearson correlations and independent samples *t*-tests assessed the associations between the variables. Assumptions of normality for continuous variables and residuals in the regression models were confirmed, as all skewness and kurtosis coefficients were within the limits proposed in the literature (West et al., 1995). In addition, the homoscedasticity assumption of residuals was confirmed via non-significant White tests, which also gage nonlinear forms of heteroscedasticity (White, 1980). Multicollinearity was also ruled out as a potential confound, as the variance inflation factors were lower than 1.5. Missing cases were less than 0.01% and thus not imputed. Finally, Common Method Variance was unlikely a serious confound (Podsakoff et al., 2012), as the single factor CFA model yielded a poor fit (χ^2^ (434) = 2386.42, *p* < 0.001; RMSEA = 0.12, *p* < 0.001, 90% *CI* (0.11, 0.12); CFI = 0.49; TLI = 0.46; SRMR = 0.11). The polytomous LCA analyses were conducted via the poLCA package in R (Linzer and Lewis, 2011). All other analyses were conducted using STATA v. 15. (StataCorp, 2015).

## Results

### Confirmatory Factor Analysis

Confirmatory Factor Analyses (CFA) verified the psychometric properties of the EI and health consciousness scales. The CFA results appear in Table 1.

**Table 1:** Unstandardized and standardized loadings for confirmatory factor analysis of emotional intelligence and health consciousness scales.

Item	Standardized factor loadings	EI Subscale (Standardized loading on EI total) *AVE* and *CR*	*M* (*SD*)
*Emotional Intelligence Scale (EI)*			
I have a good sense of why I have certain feelings.	0.79^∗∗∗^	SEA (0.68^∗∗∗^) *AVE* = 0.67 *CR* = 0.89	5.49 (1.05)
I have a good understanding of my own emotions.	0.92^∗∗∗^		
I really understand what I feel.	0.89^∗∗∗^		
I always know whether or not I am happy.	0.63^∗∗∗^		
I always know my friends’ emotions.	0.66^∗∗∗^	OEA (0.47^∗∗∗^) *AVE* = 0.60 *CR* = 0.81	5.70 (0.93)
I am a good observer of others’ emotions.	0.71^∗∗∗^		
I am sensitive to the emotions of others^a^.			
I understand the emotions of people around me.	0.92^∗∗∗^		
I always set goals and try my best to achieve them.	0.70^∗∗∗^	UOE (0.72^∗∗∗^) *AVE* = 0.60 *CR* = 0.85	5.66 (1.00)
I always tell myself I am a competent person.	0.67^∗∗∗^		
I am a self-motivated person.	0.87^∗∗∗^		
I would always encourage myself to try my best.	0.83^∗∗∗^		
I can control my temper and handle difficulties.	0.78^∗∗∗^	ROE (0.62^∗∗∗^) *AVE* = 0.73 *CR* = 0.89	5.35 (1.18)
I am quite capable of controlling my own emotions.	0.80^∗∗∗^		
I can calm down quickly when I am very angry^a^.			
I have good control over my own emotions.	0.97^∗∗∗^		
χ^2^ (70) = 88.37, *p* = 0.07; RMSEA = 0.03, *p* = 0.98, 90% CI (0.00, 0.05); CFI = 0.99; TLI = 0.99; SRMR = 0.05.			
*Health Consciousness Scale (HCS)*			
I reflect about my health a lot.	0.61^∗∗∗^	*AVE* = 0.51 *CR* = 0.86	5.39 (0.98)
I am very self-conscious about my health^a^.			
I know my inner feelings about my health.	0.79^∗∗∗^		
I am constantly examining my health.	0.70^∗∗∗^		
I am alert to changes in my health.	0.63^∗∗∗^		
I am usually aware of my health^a^.			
I am frequently aware of the state of my health.	0.77^∗∗∗^		
I notice how I feel physically through the day^a^.			
I am very involved with my health.	0.77^∗∗∗^		
χ^2^ (4) = 7.56, *p* = 0.11; RMSEA = 0.05, *p* = 0.39, 90% CI (0.00, 0.11); CFI = 0.99; TLI = 0.98; SRMR = 0.02.			

^a^*Item was eliminated due to low factor loadings (<0.60). SE, standard error; AVE, Average Variance Extracted; CR, Construct Reliability. SEA and OEA correspond to self and others’ emotional appraisal, UOE and ROE correspond to use and regulation of emotions. The measurement model including EI and HCS yielded the following goodness of fit values: χ^*2*^ (148) = 174.33, *p* = 0.07; RMSEA = 0.02, *p* = 0.99, 90 % *CI* (0.00, 0.04); CFI = 0.99; TLI = 0.99; SRMR = 0.06. Some items within each scale were paraphrased due to space limitations. ^∗∗∗^*p* < 0.001*.

As shown, the measurement model yielded adequate absolute and relative fit statistics. In both constructs the factor loadings per item were significant (*p* < 0.001), but a few items were excluded as their factor loadings were below the benchmark of 0.60 suggested in the literature (Comrey and Lee, 1992). Also, as shown the values for AVE and CR were larger than the recommended benchmarks of 0.50 and 0.70, respectively. In addition, as shown on the third column (Table 1), all subscales significantly loaded onto a higher order factor representing global or total EI. Table 2 presents the correlations, shared variance and Cronbach’s alpha estimates for each construct.

**Table 2:** Correlations, squared correlations, and internal consistency estimates.

Item	1	2	3	4	5	6
(1) SEA	α = 0.88	0.12	0.19	0.14	0.62	0.10
(2) OEA	0.34^∗∗∗^	α = 0.85	0.08	0.02	0.31	0.12
(3) UOE	0.44^∗∗∗^	0.28^∗∗∗^	α = 0.85	0.19	0.61	0.17
(4) ROE	0.37^∗∗∗^	0.13^∗^	0.44^∗∗∗^	α = 0.87	0.48	0.04
(5) EI Total	0.79^∗∗∗^	0.56^∗∗∗^	0.78^∗∗∗^	0.69^∗∗∗^	α = 0.88	0.19
(6) HCS	0.31^∗∗∗^	0.34^∗∗∗^	0.41^∗∗∗^	0.21^∗∗∗^	0.44^∗∗∗^	α = 0.84

*SEA and OEA correspond to self and others’ emotional appraisal, UOE and ROE correspond to use and regulation of emotions. Cronbach’s alpha estimates of internal consistency are presented along the diagonal, Pearson correlations appear below the diagonal and the shared variances for each construct appear above the diagonal. The AVEs per construct were: SEA = 0.67, OEA = 0.60, UOE = 0.60, ROE = 0.73, and HCS = 0.51. ^∗^*p* < 0.05, ^∗∗^*p* < 0.01, ^∗∗∗^*p* < 0.001*.

All constructs were positively correlated with each other, and the correlations between all EI subscales as well as the total EI measure and health consciousness were positive and moderate to strong. In addition, the estimates for internal consistency were above 0.80 for all constructs and in each case, the AVEs were larger than the shared variance for each pair of constructs, thus confirming discriminant validity.

### Latent Class Analysis on Health Behaviors

**FIGURE 1:**
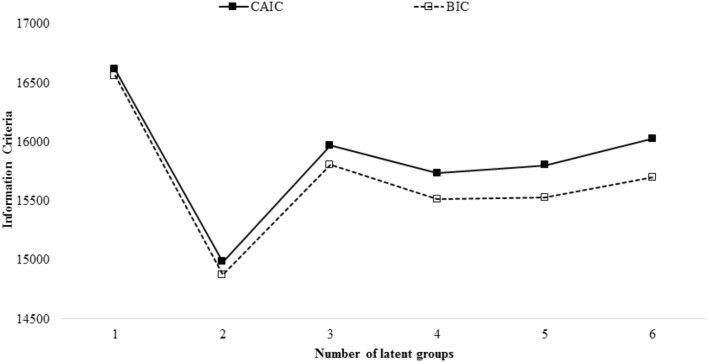
Information criteria from latent class analysis of health behaviors plotted against number of classes. BIC, Bayesian Information Criterion; CAIC, Consistent Information Criterion. The best-fitting model yields a minimum BIC and CAIC. Entropy for the two-group solution was 0.92.

To determine the number of health behavior groups that characterize the population represented by the sample, we consecutively ran the LCA model increasing the number of groups at each iteration and retaining the aforementioned information criteria (i.e., BIC and CAIC). Figure 1 presents these information criteria plotted against the respective number of LCA groups.

As shown, the two-group model minimized the information criteria and thus yielded the best-fit overall. In addition, the entropy for the two-group model was 0.92, representing clear class delineation.

Table 3 presents the probability of response for each item, conditional on group membership, as well as group comparisons in terms of sample characteristics and relevant psychometric variables.

**Table 3:** Item response probabilities of health behaviors and descriptives by latent group (*N* = 314).

	Group 1	Group 2	
	Unhealthy	Healthy	
Group Size % (*n*)	44.3 (139)	55.7 (175)	
Items *(I am often to always successful at …)*			
Eating a healthy diet (low fat and high fiber)	0.09	**0.64**	
Exercising at least 20 min daily (3× week)	0.24	**0.64**	
Flossing teeth daily	0.15	**0.56**	
Protecting skin from sun	0.32	**0.57**	
Not smoking cigarettes	**0.93**	**0.93**	
Limiting alcohol to 1 drink per day	**0.86**	**0.99**	
Taking medication as prescribed	**0.74**	**0.89**	
Cervical/prostate cancer screening (every 3 years.)	0.16	0.38	
Performing a monthly breast exam	0.07	0.28	

	***M* (*SD*)**	***M* (*SD*)**	***d***

HSC	4.89 (1.01)	5.79 (0.74)	1.02^∗∗∗^
SEA	5.31 (1.11)	5.64 (0.98)	0.32^∗∗^
OEA	5.56 (0.99)	5.82 (0.87)	0.29^∗^
UOE	5.37 (1.11)	5.88 (0.83)	0.52^∗∗∗^
ROE	5.09 (1.27)	5.54 (1.06)	0.38^∗∗∗^
EI Total	5.34 (0.79)	5.73 (0.66)	0.54^∗∗∗^
Age	21.79 (6.33)	22.07 (6.70)	0.04^n.s^
Household Income	2.37 (1.37)	2.55 (1.40)	0.13^n.s^
	% (*n*)	% (*n*)	χ^2^ (df)
Female	62.6 (87)	62.3 (109)	0.00 (1)^n.s^

*Probability of responding either “Often Successful,” “Frequently Successful” or “Always Successful” conditional on latent class or group are presented in the first panel, and those in bold are >0.5. The second panel contains descriptive statistics of the sample by health behavior group. *d*, Cohen’s *d* measure of effect size corresponding to independent samples *t*-test. HSC, Health Consciousness Scale; SEA, Self-Emotional Appraisal; OEA, Other’s Emotional Appraisal; UOE, Use of Emotion; ROE, Regulation of Emotions. EI Total is the global measure of emotional intelligence. ^∗^*p* < 0.05; ^∗∗^*p* < 0.01; ^∗∗∗^*p* < 0.001*.

As indicated, the first group (44.3%, *n* = 139) was characterized by individuals who reported not being often successful in engaging in health-promoting behaviors such as eating healthy, exercising regularly, flossing daily, and protecting their skin from the sun. The second group (55.7%, *n* = 175) was composed of individuals who were at least often successful in engaging in these health-promoting behaviors. Both groups were highly successful in taking medications as prescribed, limiting alcohol consumption and avoiding smoking cigarettes. Additionally, the two groups were equally unlikely to engage in regular cancer screening. Accordingly, we labeled the first group as *Unhealthy* and the second group as *Healthy*. In terms of sex, age and family income, the two groups were not statistically different from each other as presented at the bottom of Table 3. Yet, the *Healthy* group had higher health consciousness and EI scores than the *Unhealthy* group. These differences were from moderate to large as indicated by their respective Cohen’s *d* measure of effect size.

### Mediation Analyses

**FIGURE 2:**
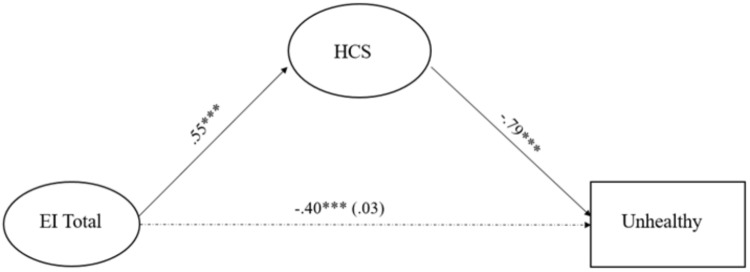
Path diagram of the relation between El global and health behaviors mediated by HCS. El Total, global measure of emotional intelligence; HCS, health consciousness scale. The model’s fit was χ^2^ (153) = 175.74, *p* = 0.10; RMSEA = 0.02, [90% CI (0.00, 0.04)], *p* = 0.99, CFI = 0.99, TLI = 0.97, SRMR = 0.05. The dotted line corresponds to total and direct effect of emotional intelligence. The total effect (HCS not included) is outside parenthesis, and the direct effect, computed when HCS was included in the model, is in parenthesis. ^∗∗∗^*p <* 0.001.

**FIGURE 3:**
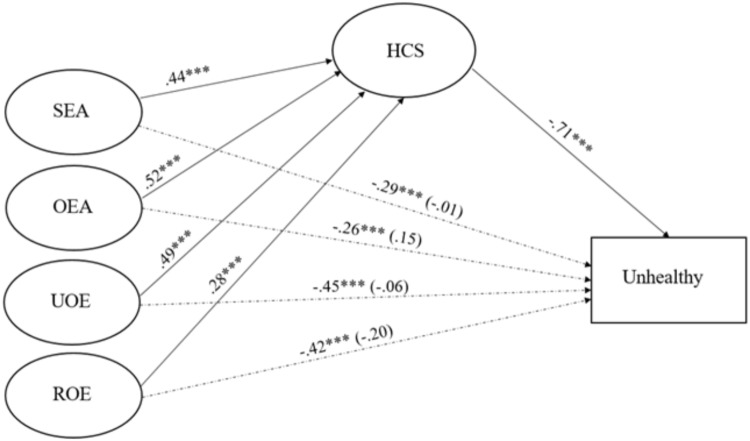
Paths for the relation between SEA, OEA_;_ UOE and ROE and health behaviors mediated by HCS. SEA, Self-Emotional Appraisal; OEA, Other’s Emotional Appraisal; UOE, Use of Emotion; ROE, Regulation of Emotions; HCS, health consciousness scale. Model’s fit was χ^2^ (168) = 199.08, *p* = 0.05, RMSEA = 0.02, 90% CI (0.00, 0.04), *p* = 0.99), CFI = 0.99, TLI = 0.99, SRMR = 0.05. The dotted lines correspond to total and direct effects of the four dimensions of emotional intelligence. Direct effects, computed when HCS was in the model, are in parenthesis. ^∗∗∗^*p* < 0.001.

Mediation analyses assessed the indirect effect of health consciousness on the relation between EI and the probability of belonging to the *Unhealthy* group, relative to the *Healthy* group. Specifically, we conducted two mediation models. The first model used the global measure of EI, and the second model used the four EI subscales (i.e., SEA, OEA, UOE, and ROE) as antecedents. Every model included sex, age, and family income as covariates, although none of them significantly related to the outcomes. The results are presented in Table 4, and depicted in Figures 2, 3.

**Table 4:** Direct, total, indirect effects and effect sizes of the relation between emotional intelligence and health-related behaviors mediated by health consciousness.

Antecedent	Direct effect (HSC included)	Total effect (HCS not included)	Indirect effect normal-based 95% confidence interval	Partially standardized indirect effect	Completely standardized indirect effect
EI Total	-0.03	-0.40^∗∗∗^	-0.44 (-0.61, -0.26)^∗^	-0.88	-0.66
SEA	0.06	-0.29^∗∗∗^	-0.35 (-0.51, -0.19)^∗^	-0.70	-0.74
OEA	0.15	-0.26^∗∗∗^	-0.41 (-0.57, -0.25)^∗^	-0.82	-0.76
UOE	-0.06	-0.45^∗∗∗^	-0.39 (-0.54, -0.24)^∗^	-0.78	-0.78
ROE	-0.20	-0.42^∗∗∗^	-0.22 (-0.28, -0.06)^∗^	-0.44	-0.52

*CI, Confidence Interval; EI, Emotional Intelligence; SEA, Self-Emotional Appraisal; OEA, Other’s Emotional Appraisal; UOE, Use of Emotion; ROE, Regulation of Emotions; HCS, health consciousness scale. The Healthy group is the referent health-related behavior group. EI Total is the global measure of emotional intelligence. Indirect effects and 95% CI were obtained via 5,000 bootstrapped replications. Goodness of fit statistics for the model using EI total: χ^*2*^ (153) = 175.74, *p* = 0.10; RMSEA = 0.02, 90% CI (0.00, 0.04), *p* = 0.99, CFI = 0.99, TLI = 0.97, SRMR = 0.05. Goodness of fit statistics for the model using the four subscales for EI: χ^*2*^ (168) = 199.08, *p* = 0.05; RMSEA = 0.02, 90% CI (0.00, 0.04), *p* = 0.99, CFI = 0.99, TLI = 0.99, SRMR = 0.05. ^∗^*p* < 0.05, ^∗∗^*p* < 0.01, ^∗∗∗^*p* < 0.001*.

In every instance EI was significantly related to decreases in the likelihood of belonging to the *Unhealthy* group relative to the *Healthy* group, and related to increments in health consciousness. Health consciousness was in turn related to decreases in the likelihood of belonging to the *Unhealthy* group relative to the *Healthy* group. Upon adding health consciousness to the model, the effect of EI decreased in magnitude, and was no longer significant. As indicated in the 4th column of Table 4, the indirect effect of health consciousness was significant, and according to both measures of effect size, the effect was large. Both models yielded sound goodness of fit statistics as indicated at the bottom of Table 4 and Figures 2, 3.

## Discussion

This study assessed health-related behaviors within a large sample of young adults, and gaged the roles of emotional intelligence and health consciousness in predicting such. Latent class analyses identified two health-behavior groups that resemble the two broad dimensions of preventive and risk-taking behaviors observed elsewhere (Vickers et al., 1990) and thus highlight the external validity of the findings. Namely, our young adult sample was divided into two distinct health-behavior groupings: An unhealthy group (not successful at eating healthy, exercising regularly, flossing daily, and protecting their skin from the sun) and a healthy group (successful at eating healthy, exercising regularly, flossing daily, and protecting their skin from the sun). While the two groups were similar in terms of socio-demographic characteristics, the healthy group endorsed on average higher levels of health consciousness than the unhealthy group; a result that is in agreement with the literature, and our first hypothesis. More specifically, studies have shown that the degree to which individuals are concerned about their health is a strong indicator of the extent to which they will engage in health promoting behaviors, such as fitness, nutrition, and others (Kraft and Goodell, 1993; Forthofer and Bryant, 2000). Accordingly, and as observed herein, individuals who engage in health-responsible or promoting behaviors are expected to have higher levels of health consciousness than individuals who do not engage in such health-promoting behaviors.

Also, as hypothesized the healthy group had higher levels of emotional intelligence than the unhealthy group. These findings are consistent with prior evidence indicating that individuals with high emotional competencies have the propensity to engage in healthy behaviors (Johnson et al., 2009; Zeidner et al., 2012; Mikolajczak et al., 2015). Given the multitude of studies indicating that health behaviors are strongly connected to long-term physical health (e.g., Colditz et al., 2000; Chiuve et al., 2012), our findings provide additional insights building on results from meta-analytic studies documenting emotional intelligence as predictor of improved physical health (Schutte et al., 2007; Martins et al., 2010), and further highlight the importance of understanding the mechanisms that explain the emotional intelligence to health behaviors relation. Specifically, in this article we confirmed health consciousness as a conduit explaining such relation, as structural equation models yielded significant indirect paths by way of health consciousness in the relations between emotional intelligence and health behaviors, holding constant age, sex, and income. These findings expand our knowledge on the well-documented positive effect of emotional intelligence on health (Schutte et al., 2007; Martins et al., 2010) by suggesting that emotional intelligence assists in the improvement of one’s health consciousness, and consequently participation in behaviors that predict long-term physical health.

Importantly, all dimensions of emotional intelligence including appraisal (self and others’), use and regulation of emotions, were relevant for reductions in the likelihood of engaging in unhealthy behaviors compared to healthy ones by way of improvements in health consciousness. These findings add to the literature seeking to identify the unique contribution of different dimensions of emotional intelligence on health-related outcomes (Fernández-Abascal and Martín-Díaz, 2015). Specifically, our findings highlight the additive value of each dimension of emotional intelligence, as operationalized by Wong and Law (2002) toward understanding the mechanism that explains the connection between emotional capabilities and health promoting behaviors among young adults. These findings suggest that emotional intelligence, in all its dimensions, is a precursor to increases in one’s health awareness, resulting in acquiescence to healthy behaviors. In combination, our findings suggest that educational programs aiming to improve health behaviors among young adults should include emotional intelligence training in their curriculum, as such training may help develop intrinsic health awareness and the willingness to promote health. Yet, there is a need for additional research to investigate these claims more rigorously.

Particularly, a key feature of emotional intelligence is the capacity for emotion regulation (Mayer and Salovey, 1995; Peña-Sarrionandia et al., 2015), which concerns the process by which individuals regulate their emotions (Peña-Sarrionandia et al., 2015) and is an important predictor of engaging in effective behaviors for managing stress (Compas et al., 2014; Kober, 2014). Along these lines our findings, while in agreement with studies indicating a negative association between emotional intelligence and health-impairing coping behaviors (Mikolajczak et al., 2009; Espinosa et al., 2018), raise awareness about the need to investigate emotion regulation strategies as additional mechanisms explaining the connection between emotional intelligence and health behaviors, particularly as they relate to health consciousness. In the same vein, our study raises awareness about the need to identify the extent to which other salient predictors of health behaviors relate to emotional intelligence and health consciousness. In particular, researchers have highlighted genetic factors as important predictors of smoking, healthy eating, physical fitness and other behaviors (Bulik, 2004; Sutton, 2005; Chomistek et al., 2013). The interrelation between genetic predispositions, emotional intelligence and health consciousness in predicting health behaviors is unknown, yet necessary for developing effective and comprehensive approaches to improve health-related behaviors among young adults.

This study is not without limitations. First, due to our use of self-report measures we cannot rule out social desirability bias influencing the findings. Second, the study employed a cross-sectional design, which prevents us from making causal interpretations. Future studies should consider a longitudinal design that would allow for robust causal interpretations, and include measures of social desirability as covariates. Along these lines, while the measure of emotional intelligence used in this study has been identified in the literature as a reliable construct of emotional self-efficacy (Pérez et al., 2005) whose first-order factors are distinct, but correlated dimensions defining the focal construct of emotional intelligence (Walter et al., 2011), future research should also include more objective measures of emotional intelligence to address any concerns related to the use of self-report measures. Third, both health groups exhibited equally poor cancer-screening behaviors, which prevents us from generalizing the findings to these specific disease prevention approaches. On a similar vein, the two groups were highly unlikely to use substances such as nicotine, and alcohol, which is atypical of college students (Center for Behavioral Health Statistics and Quality, 2015; Skidmore et al., 2016), and thus may highlight a unique aspect of our sample that limits generalization to other young adult samples. Future studies should cast a wider net to recruit participants by perhaps selecting multiple sampling units. Finally, our study did not include an exhaustive list of health behaviors, thus limiting the findings to the health behaviors gauged herein. Future studies should include additional types of behaviors such as risky sexual behaviors and illicit drug use.

Despite these limitations, our study provides new evidence highlighting the mediating role of health consciousness in the relation between emotional intelligence and health behaviors. Given the predictive role of health behaviors during early adulthood on health outcomes in old age, the results are relevant for educators and policymakers, as they present a mechanism linking young adults’ personal characteristics to behaviors that reduce the risk of chronic illness later in life.

## Conclusion

This study determined the types of health behaviors that characterize our young adult population as well as the relations between such health behaviors, health consciousness and emotional intelligence. Furthermore, this study tested health consciousness as mediator of the relation between emotional intelligence and health behaviors. The results indicate that health behaviors among young individuals can be viewed as a dichotomy of healthy and unhealthy behaviors. Individuals whose behaviors qualify as healthy tend to have higher levels of emotional intelligence than those whose behaviors are characterized as unhealthy. In our findings, health consciousness exerted an intervening pathway that explained the relation between emotional intelligence and health behaviors. Namely, individuals with low emotional intelligence also had low health consciousness, and thus a higher propensity to engage in unhealthy behaviors relative to individuals with high emotional intelligence. Accordingly, programs seeking to improve health behaviors among young adults should consider the inclusion of emotional intelligence training within the curriculum, as improvements in emotional competencies are likely to influence the development of health consciousness and accordingly improve behaviors that promote health and well-being.

## Ethics Statement

This study was carried out in accordance with the recommendations of the Institutional Review Board of the City College of New York with written informed consent from all participants. All participants gave written informed consent in accordance with the Declaration of Helsinki. The protocol was approved by the Institutional Review Board of the City College of New York.

## Data Availability Statement

The raw and de-identified data supporting the conclusions of the manuscript will be made available by the authors, without undue reservation, to any qualified researcher.

## Author Contributions

Both authors, AE and SK-M, contributed to the project’s design, administration, survey creation, interpretation of findings, writing, and revising of the manuscript. AE contributed to the collection and analysis of data. Both authors approved the final version of this manuscript.

## Conflict of Interest Statement

The authors declare that the research was conducted in the absence of any commercial or financial relationships that could be construed as a potential conflict of interest.
